# Automated design of dynamic programming schemes for RNA folding with pseudoknots

**DOI:** 10.1186/s13015-023-00229-z

**Published:** 2023-12-01

**Authors:** Bertrand Marchand, Sebastian Will, Sarah J. Berkemer, Yann Ponty, Laurent Bulteau

**Affiliations:** 1 LIX (UMR 7161), Ecole Polytechnique, Institut Polytechnique de Paris, Palaiseau, France; 2 LIGM, CNRS, University Gustave Eiffel, F77454 Marne-la-Vallée, France; 3 Earth-Life Science Institute, Tokyo Institute of Technology 2–12-1-I7E-318, Ookayama, Tokyo 152–8550 Japan

**Keywords:** Pseudoknots, RNA folding, Tree Decomposition, Treewidth

## Abstract

Although RNA secondary structure prediction is a textbook application of dynamic programming (DP) and routine task in RNA structure analysis, it remains challenging whenever pseudoknots come into play. Since the prediction of pseudoknotted structures by minimizing (realistically modelled) energy is NP-hard, specialized algorithms have been proposed for restricted conformation classes that capture the most frequently observed configurations. To achieve good performance, these methods rely on specific and carefully hand-crafted DP schemes. In contrast, we generalize and fully automatize the design of DP pseudoknot prediction algorithms. For this purpose, we formalize the problem of designing DP algorithms for an (infinite) class of conformations, modeled by (a finite number of) fatgraphs, and automatically build DP schemes minimizing their algorithmic complexity. We propose an algorithm for the problem, based on the tree-decomposition of a well-chosen representative structure, which we simplify and reinterpret as a DP scheme. The algorithm is fixed-parameter tractable for the treewidth *tw* of the fatgraph, and its output represents a ${\mathcal {O}}(n^{tw+1})$ algorithm (and even possibly ${\mathcal {O}}(n^{tw})$ in simple energy models) for predicting the MFE folding of an RNA of length *n*. We demonstrate, for the most common pseudoknot classes, that our automatically generated algorithms achieve the same complexities as reported in the literature for hand-crafted schemes. Our framework supports general energy models, partition function computations, recursive substructures and partial folding, and could pave the way for algebraic dynamic programming beyond the context-free case.

## Introduction

The function of non-coding RNAs is, to a large extent, determined by their structure. Structure prediction algorithms therefore play a crucial role in biomedical and pharmaceutical applications. The basis to determine more complex 3D structures of RNA molecules is set by first accurately predicting their 2D or secondary structures. There exist various RNA folding algorithms that predict an optimal secondary structure as *minimum free energy structure* of the given RNA sequence in suitable thermodynamic models. In the most frequently used methods, this optimization is performed efficiently by a dynamic programming (DP) algorithm, e.g. mfold [1], RNAfold [2], RNAstructure [3]. A recent alternative to predictions based on experimentally determined energy parameters are machine learning approaches that train models on known secondary structures, e.g., CONTRAfold [4], ContextFold [5], MXfold2 [6].

**Fig. 1:**
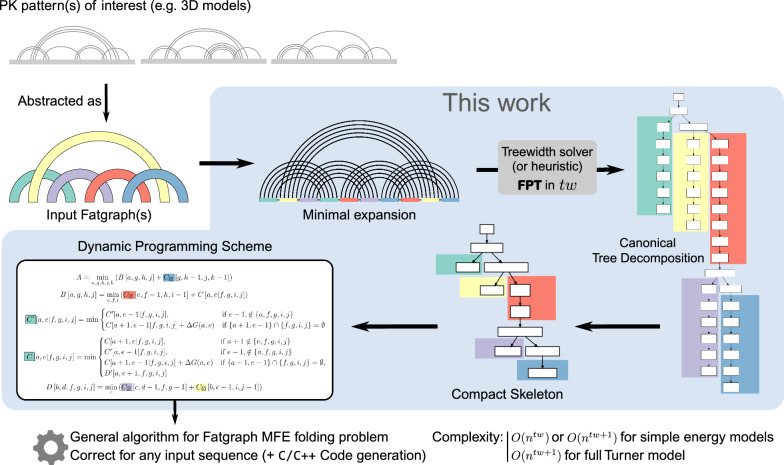
Given a finite number of arbitrary fatgraphs, a dynamic programming scheme for folding (restricted to the family of structures specified by the fatgraphs) is derived from canonical tree decompositions of minimal representative expansions of the helices, for each fatgraph. The workflow gives an overview of the steps of the algorithm. Each step is described in more details in the subsequent sections and figures: see Fig. 2 for fatgraphs, Fig. 5 and Sect. "Minimal representative expansion of a fatgraph"  for a detailed version of the canonical tree decomposition, Fig. 8 for a detailed view of the compact skeleton of the tree decomposition

However, the most frequently used algorithms (including all of the above ones) optimize solely over pseudoknot-free structures [7], which do not contain crossing base pairs. Although pseudoknots (PK) appear in many RNA secondary structures, they have been omitted by initial prediction algorithms due to their computational complexity [8], and the difficulty to score individual conformations [9]. Nevertheless, many algorithms have been proposed to predict at least certain pseudoknots. These methods are either based on exact DP algorithms such as pknots-RE [10], NUPACK [11], gfold [12], Knotty [13] or they use heuristics that don’t guarantee exact solutions, e.g., HotKnots [14], IPknot [6, 15], Hfold [16].

Owing to the hardness of PK prediction, efficient exact DP algorithms are necessarily restricted to certain categories of pseudoknotted structures. The underlying DP schemes are designed manually, guided by design to either i) support structures that are frequently observed in experimentally resolved structures (declarative categories); or ii) support the largest possible set of conformations, while remaining within a certain complexity (complexity-driven). For most categories, essentially declarative ones, there exists one or several helix arrangements, either observed in experimentally-determined structures or implicitly characterized by graph-theoretical properties (3-non-crossing [17], topologically bounded [12]) that need to be captured. A detailed overview of pseudoknot categories is given in [18]. Similar situations occur for RNA-RNA interactions [19], possibly including several RNA molecules. Interestingly, when more than two RNA strands are considered, existing algorithms restrict the joint conformation to crossing-free interactions [20], further motivating an, ideally-automated, design of algorithms beyond the case of pseudoknot-free secondary structures.

The paradigm of tree decompositions (TD) represents an appealing candidate for automating such a design task. TDs organize the vertices of a graph into a tree-like structure that represents all vertices and edges, augmented with a notion of consistency. A TD can then be re-interpreted as DP schemes for a wealth of graphs problems involving local constraints (coloring, independent sets, covers...) [21] and complex pattern matching problems in Bioinformatics [22]. The complexities of such exact algorithms are typically exponential on a parameter called the treewidth, which can be minimized to obtain an optimal TD in time only exponential on the min treewidth itself [23]. However, TD-based approaches typically start from a single input graph, whereas folding prediction requires DP schemes that generalize to collections of structures of unbounded cardinalities. This led us to the following question, at the foundation of this work:

*Can tree decompositions be used to infer structure prediction algorithms that work for entire classes of conformations?*

In this work, we answer positively to that question. We consider popular classes of pseudoknotted structures, described as fatgraphs [12, 24–26], an abstraction of RNA conformations related to RNA shapes [27] or shadows [12, 17]. We formalize the principles underlying the design of DP folding algorithms including pseudoknots and, at the same time, give a formulation of the computational problem corresponding to the design of DP algorithms. We show how to leverage tree-decompositions, computed on a minimal expansion of the input fatgraph, to automatically derive DP schemes that use as little indices as possible. Our methodology leads to a generalization of algorithms underlying LiCoRNA [22] and gfold [12] and represents a parameterized algorithm based on the treewidth (*tw*) of the underlying fatgraph. For example, our method automatically derives optimally efficient recursions of a gfold-like prediction algorithm covering the four pseudoknot types of 1-structures (cf Table 1) Moreover, it enables highly complex implementations, like a prediction algorithm for 2-structures. Notably, this was never implemented for gfold, since it requires the generation of recursions for 3472 fatgraphs—virtually impossible to conduct “by hand”.

In Sect. "Definitions and main result", we state our problem and define its input structure abstraction, the fatgraph. Then, we describe helix expansions of the fatgraph and their tree decompositions (Sect. "Minimal representative expansion of a fatgraph"). By minimal helix expansions and a derivation of the tree decomposition to its canonical form, we automatically derive a DP scheme for the folding of pseudoknotted structures (Sect. "Interpreting the tree decomposition of a fatgraph expansion as a DP algorithm"). The following result is the main result of our papering a number of indices equal to the treewidth. Figure 1 outlines the fundamental algorithm. Section "Extensions" discusses extensions to combine multiple fatgraphs, include recursive substructure, and cover realistic energy models. Section "Automated (re-)design of algorithms for specific pseudoknot classes" discusses the application of our methods to the design of concrete pseudoknot folding algorithms. We demonstrate the re-design of gfold for 1-structures, as well as the novel design of 2-structure prediction and interesting novel algorithms between 1- and 2-structures (e.g. predicting 5-chains in $O(n^7)$).

## Definitions and main result

We define an *RNA sequence* *S* as a word of length *n* over the nucleotides *A*, *C*, *G* and *U*; moreover an *RNA secondary structure* (potentially, with pseudoknots) $\omega$ of *S* as a set of *base pairs* (*i*, *j*) between sequence positions *i* and *j* (in 1, ..., *n*), such that there is at most one base pair incident to each position. A *diagram* is a graph of nodes 1,...,*n* (the positions), connecting consecutive positions by directed edges $(i,i+1)$ and moreover connecting positions by arcs, visualizing the *arc-annotation* of the sequence. Typically this is represented drawing the backbone linearly and the arcs on top. RNA secondary structures are naturally interpreted as diagrams.

One of our central concerns is the crossing configuration of arcs in a diagram. We define two arcs (*i*, *j*) and $(i',j')$ in a diagram as *crossing* iff $i<i'<j<j'$ or $i'<i<j'<j$. Naturally, this leads to the notion of a conflict graph consisting of all the arcs of a diagram and connecting crossing arcs by a conflict edge. Given a potentially conflicted set of base pairs, the associated *RNA structure graph* is the diagram consisting of one vertex per nucleotide, backbone links, and one arc per base pair.

**Fig. 2:**
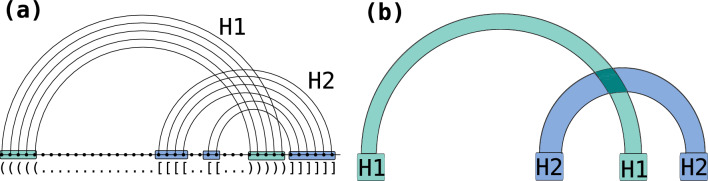
**a** Diagram of a secondary structure with two crossing helices (H1 green, H2 blue). **b** fatgraph corresponding to the above structure such that helices are collapsed into bands and form the shadow of the structure

A* fatgraph* [12, 24–26] is an abstraction of a family of pseudoknotted RNA structures displaying a specific conflict structure. It is typically represented as a *band diagram* (see Figs. 1 and 2), in which each band may represent a *helix* of arbitrary size, including bulges. An arc-annotation is said to be an *expansion* of a fatgraph if collapsing nested arcs and contracting isolated bases yields the band diagram of a fatgraph. Given a finite number of fatgraphs, we say a structure is a *recursive expansion* of these fatgraphs if decomposing the structure into conflict-connected components, collapsing nested arcs and contracting isolated bases only yields members of the given fatgraph set. For the purpose of this presentation (where we do not explicitly study structure topology), we moreover identify fatgraphs with their diagrams.

To make the connection to gfold [12] explicit, recursive expansions of fatgraphs are equivalently understood in terms of the shadows of a structure. The shadow of an RNA structure (or equivalently, its diagram) is defined in [12] as the diagram obtained by, firstly, removing all unpaired bases and non-crossing structures and, secondly, contracting all stacks (i.e. pairs of arcs between directly consecutive positions) to single arcs. Then, the class of recursive expansions of a set of input fatgraphs $\Gamma$ is the class of structures, where the shadows of their conflict-connected components are in $\Gamma$.

In this paper, we consider a class of RNA folding problems in which the search space is restricted to recursive expansions of a user-specified finite set of fatgraphs. For the sake of simplicity, we first describe minimizing energy in a simple free-energy model ${\mathcal {E}}$, where the energy of a sequence/structure is obtained by summing the contributions of individual base pairs; moreover, we present the method initially without recursive insertions. Only later, in Sect. "Extensions", we extend to the full problem in realistic energy models.

### Definition 1

(Fatgraph MFE folding problem) **Input:** Collection of fatgraphs $\gamma _1,\dots ,\gamma _p$, sequence *S*

**Output:** Minimum Free Energy (MFE) arc-annotation for *S* according to a free-energy model ${\mathcal {E}}$, restricting the search to recursive expansions of the input fatgraphs.

Specifically, we solve the problem of automatic design of such pseudoknot prediction algorithms based on an input set of fatgraphs.

### Definition 2

(Fatgraph algorithm design problem) **Input:** Collection of fatgraphs $\gamma _1,\dots ,\gamma _p$

**Output:** A Dynamic-Programming algorithm that, given any sequence *S*, solves the Fatgraph MFE folding problem over $\gamma _1,\dots ,\gamma _p$ and *S*.

Defining the treewidth of a fatgraph as the treewidth of its minimal expansion (see Sect. "Helices of length 5 are sufficient to obtain generalizable tree decompositions"), our main result, stated in Algorithm 1, is the existence of an effective algorithm for the Fatgraph MFE-folding problem, parameterized by the maximum treewidth *tw* of the input fatgraphs. Using parameterized algorithmics terminology [28], it consists of an FPT (Fixed-Parameter Tractable) preprocessing of the input fatgraphs, yielding an XP (Slicewise-Polynomial) dynamic-programming algorithm accepting any input sequence and solving the Fatgraph MFE folding problem (see Fig. 1). In a nutshell, an algorithm is FPT in a parameter *k* is its run-time is of the form $O(f(k)\cdot n^c)$, for *c* a constant and *f* a computable (typically super-exponential) function. On the other hand, it is XP if its run-time is of the form $f(k)\cdot n^{g(k)}$ for two computable functions *f*, *g*. Both yield polynomial algorithms for a fixed value of *k*. More details about the parameterized complexity classes XP and FPT can be found in [28].

**Figure:**
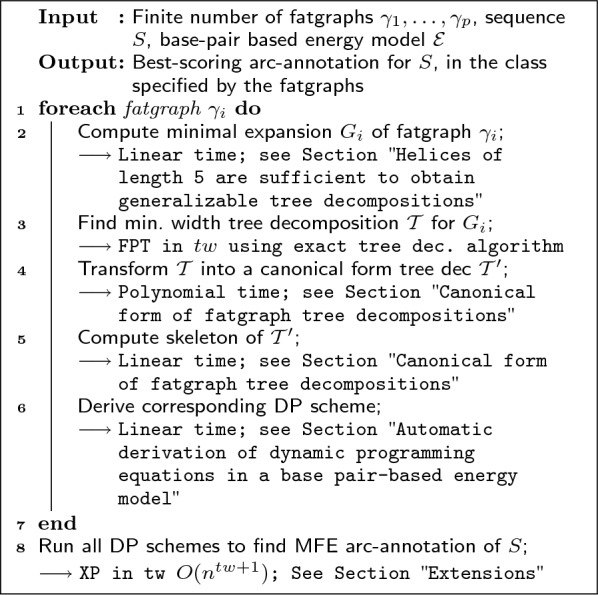
**Algorithm 1:** Pseudocode for the recursive fatgraph folding problem

The following result is the main result of our paper. A refined version is Theorem 4 in Sect. "Complexity analysis".

### Theorem 1

(Main result) Algorithm 1 solves the fatgraph folding problem in $O(n^{tw+1})$, where *tw* is the maximum treewidth of the input fatgraphs.

As detailed with Theorem 4, the complexity can also be $O(n^{tw})$ in certain cases, depending on the choice of energy model and the fatgraphs under consideration.

Since the number of indices used by the DP equation is minimized, the resulting complexities could be seen as optimal within a family of simple DP algorithms. However, a characterization of such a non-trivial family of algorithms would be beyond the scope of this work, and we leave formal proofs of optimality to future work, as briefly discussed in Sect “Conclusions and discussion”.

## Minimal representative expansion of a fatgraph

Our approach builds on the concept of tree decomposition, which we want to leverage to derive decomposition strategies within dynamic programming (DP) schemes. A key challenge is in the fact that tree decompositions are computed for concrete graphs, whereas our objective is to find an algorithm whose search space includes all possible recursive expansions of an input fatgraph.

Fortunately, we find that expanding every helix of a fatgraph to length 5 (i.e. 5 nested base pairs) yields a graph which is representative of the fatgraph. Namely, its optimal *tree decomposition*, having treewidth *tw*, trivially generalizes into a tree decomposition for any further expansion, retaining treewidth *tw*. This tree decomposition can finally be reinterpreted into a DP scheme that exactly solves the MFE folding problem in ${\mathcal {O}}(n^{tw+1})$ complexity (and sometimes even ${\mathcal {O}}(n^{tw})$ for simple energy models).

### Treewidth and tree decompositions

#### Definition 3

A tree decomposition ${\mathcal {T}}=(T,\{X_i\}_{i\in V(T)})$ of a graph $G=(V,E)$ is a tree of subsets of vertices of *G*, called bags, verifying the following conditions:

- $\forall u\in V$ $\exists i\in V(T)$ such that $u\in X_i$. (representing vertices)
- $\forall (u,v)\in E$ $\exists i\in V(T)$ such that $\{u,v\}\subset X_i$.(representing edges)
- $T_u=\{i\in V(T)\mid u\in X_i\}$ must be connected. (vertex subtree property)

The *width* of a tree decomposition is the size of its biggest bag minus one, i.e. $\max _{i\in V(T)}|X_i|-1$. The *treewidth* of a graph *G* is then the minimum possible width of a tree decomposition of *G*. Intuitively, the lower the treewidth, the closer *G* is to being a tree. Treewidth is NP-hard to compute [29], but fixed-parameter tractable (FPT): there is a $O(f(w)\cdot n)$ algorithm [23] deciding whether $tw(G)\le w$ given *G*. More details regarding the fixed-parameter tractability and theoretical aspects of treewidth can be found in [28]. Many polynomial heuristics are also known to yield reasonable results [30], and optimized exact solvers have been developed [31, 32]. Notoriously, a wide variety of hard computational problems can be solved efficiently when restricted to graphs of bounded treewidth [21, 28], including in bioinfomatics [22, 33, 34]. Such is the case of pseudoknotted structure-sequence alignment, using the algorithm presented in [22]. The method presented in this paper can actually be seen as a generalization of this algorithm, allowing to perform “pseudoknotted *motif*-sequence alignment”, with a motif describing a family of structures.

We will rely in the remainder of this section on some well known-properties for treewidth, which we recall here. First, taking any *minor* of *G* [35], i.e. performing any sequence or edge contractions, edge deletions and vertex deletions on *G* can only lower the treewidth. Second, degree-2 vertices can be contracted into their neighbors without changing the treewidth, as quickly stated below. This implies in particular that any bulge in a helix of an RNA structure graph is inconsequential with respect to treewidth.

#### Proposition 1

If *u* is a degree-2 vertex of *G* with neighbors $\{v,w\}$, and $G_{v\leftarrow u}$ is the graph obtained by contracting *u* into *v* in *G* then $tw(G)=tw(G_{v\leftarrow u})$

#### Proof

To start with, $G_{v\leftarrow u}$ is a minor of *G*, therefore $tw(G_{v\leftarrow u})\le tw(G)$. Then, given an optimal tree decomposition ${\mathcal {T}}$ for $G_{v\leftarrow u}$, since (*v*, *w*) is an edge of this graph, there has to be a bag *X* containing both vertices. If $tw(G_{v\leftarrow u})=1$, then $X=\{v,w\}$ and can be split into two bags $\{v,u\}$ and $\{u,w\}$ to obtain a tree decomposition for *G*. If $tw(G_{v\leftarrow u})\ge 2$, then we can simply connect a new bag $\{u,v,w\}$ and connect it to *X* to obtain again a valid tree decomposition for *G* of the same width. Therefore $tw(G)\le tw(G_{v\leftarrow u})$ and we have the equality. $\square$

Then, we import from [36] an inequality valid for any *separator* of *G*. A *separator* is a subset *S* of vertices of *G* such that $G\setminus S$ is composed of at least 2 conected components. This set of connected components obtained by removing *S* in *G* is denoted ${\mathcal {C}}_G(S)$. We then have:

#### Proposition 2

If *S* is a separator of *G*, then

$$\begin{aligned} tw(G) \le \max _{C\in {\mathcal {C}}_G(S)} tw(G[C\cup \texttt{clique}(S)]) \end{aligned}$$

with $G[C\cup \texttt{clique}(S)]$ the subgraph of *G* induced by $C\cup S$ augmented by edges making *S* a clique. In case of equality, we say that *S* is safe.

#### Proof

Consider, for each $C\in {\mathcal {C}}_G(S)$, a tree decomposition ${\mathcal {T}}_C$ of $G[C\cup \texttt{clique}(S)]$. Since these graphs contain *S* as a clique, each ${\mathcal {T}}_C$ must have a bag $X_C$ containing *S* entirely. Consider now the following tree decomposition for *G*: make a bag out of *S*, and connect $X_C$ for each *C* to it. The resulting tree decomposition is valid for *G*, and its width is the left-hand-side of the inequality. $\square$

Conversely, given two adjacent bags *X* and *Y* in a tree decomposition ${\mathcal {T}}$, unless all vertices on the “X-side” of the tree decomposition are also present in the *Y*-side (or the opposite), $X\cap Y$ is a separator of *G*. Formally, given (*X*, *Y*) an edge of a tree decomposition ${\mathcal {T}}$, the *X*-side of ${\mathcal {T}}$ is the connected component of ${\mathcal {T}}$ containing *X* obtained when removing (*X*, *Y*).

To write down the proofs of the following section in a smoother fashion, we restrict (w.l.o.g) tree decompositions to be such that any intersection of two adjacent bags is a *minimal* separator of the graph. The existence of optimal decompositions with these property is easily seen when defining tree decompositions in terms of *triangulations* and *chordal graphs* [31, 37]. In this framework, the treewidth of a graph *G* is the minimum possible maximum clique size in a chordal completion of *G*. The bags of the decomposition are the maximal cliques of the chordal completion (“clique-tree”), and intersections of adjacent bags are minimal separators. For completeness, we formulate this result in the following proposition:

#### Proposition 3

Given a graph *G*, there always exists an optimal tree decomposition such that, for any two adjacent bags *X* and *Y*:

1. $X\cap Y$ is a minimal separator of *G*.
2. $$|X\cap Y|\le tw(G)$$

#### Proof

Denoting $\omega (H)$ the maximum clique size of a graph *H*, we have [37]:

$$\begin{aligned} tw(G)=\underset{H\text { chordal completion of }G}{\min } \omega (H) \end{aligned}$$

The tree decomposition corresponding to a particular chordal completion *H* of *G* is the “clique-tree” of *H*. Bag intersections are then minimal separators of *G* (item 1), and no two bags contain exactly the same vertices (hence item 2). We refer the reader to [37] for full definitions and justifications. $\square$

### Helices of length 5 are sufficient to obtain generalizable tree decompositions

**Fig. 3:**
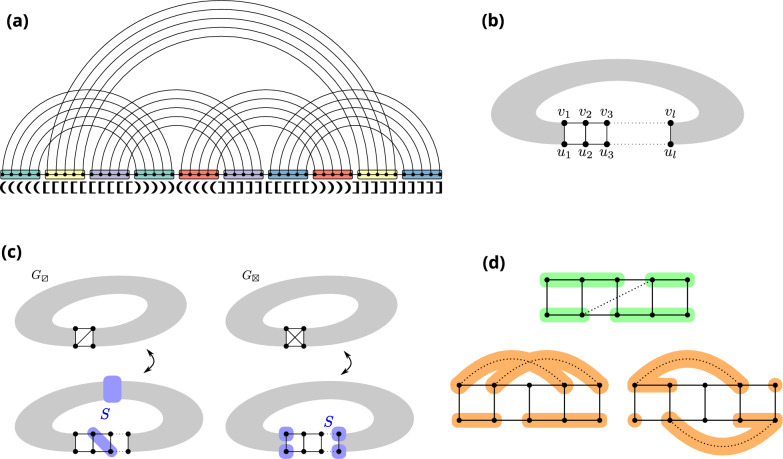
**a** minimal expansion of a fatgraph, with every helix of length 5, and no unpaired base. The associated graph consists of one vertex per base, and one edge per base pair and backbone link. **b** A helix of length *l* in an RNA graph, as per the latter definition. **c** Given a helix in a graph *G*, the treewidth of *G* is either equal to $tw(G_\boxtimes )$ or $tw(G_\boxslash )$. Each case is associated with a type of *separator* that can be used to extend the helix, or insert bulges, without changing the treewidth. (d) The dotted line represents a “hop-edge” which, if represented in a given tree decomposition of *G*, can be used to obtain $G_\boxtimes$ as a minor of *G*, showing that the helix is in the “clique” case

Given an RNA graph (with one vertex per nucleotide and one edge per base pair and backbone link, see Fig. 3a, we call *perfect helix* a set of directly nested base pairs, resulting in the subgraph depicted on Fig. 3b. We call the number of nested base pairs its *length*, and denote it with *l*. With a slight abuse of language, we call such a subgraph a *helix*, even for general graphs.

Throughout the remainder of the article, helices will be often proven to be replaceable, as a subgraph, by one of two small graphs on 4 vertices. These two graphs are the clique on 4 vertices and a 4-cycle augmented with one (and only one) of the possible two chords. To simplify the exposition, we simply denote them by $\boxtimes$ and $\boxslash$.

One situation where $\boxtimes$ will appear is when we prove that, sometimes, the 4 extremities can be connected into a clique without loss of generality. The graph we obtain, an helix closed by a clique, has treewidth 4, which will be an important threshold in our structural results below. We state this fact in the following lemma. Let us denote by $H_l^{*}$ the graph corresponding to a helix of length *l*, with the extremities connected as a clique.

#### Lemma 1

For $l=2$, $tw(H_l^{*})=3$, while for $l\ge 3$, $tw(H_l^{*})=4$.

#### Proof

For $l=2$, $H_l^{*}$ is simply the clique on 4 vertices, and which has a width of 3. For $l\ge 3$, a clique on 5 vertices can be obtained as a minor by contracting the internal part of the helix to one vertex, which ends up being connected to all 4 extremities, which already form a clique. Therefore, $tw(H_l^{*})\ge 4$. To obtain the equality, we recursively build a tree decomposition of width $\le 4$, starting with $l=2$ which we already described. Given a tree decomposition of width $\le 4$ for $H_{l}^{*}$, there has to be a bag *X* containing all 4 extremities $\{u_1,v_1,u_l,v_l\}$ (see Fig. 3b). We introduce two new bags: $X'=\{u_1,v_1,u_l,v_l,v_{l+1}\}$ introducing a new vertex $v_{l+1}$, and $X''=\{u_1,v_1,u_l,v_{l+1},u_{l+1}\}$ introducing $u_{l+1}$. We connect $X'$ to *X* and $X''$ to $X'$. By doing so, we respect the subtree connectivity property for all involved vertices, and build a tree decomposition capable of representing $H_{l+1}^{*}$. $\square$

Our main structural result is to show that the treewidth of a graph *G* does not increase when extending a helix past a length of 5. Its proof relies on the following inequality, involving the graphs $G_\boxtimes$ and $G_\boxslash$, obtained from *G* by replacing a helix *H* with either $\boxtimes$ or $\boxslash$, (see Fig. 3c).

#### Lemma 2

Given a graph *G* and a helix *H* of length $l\ge 3$ in *G*, we have:

$$\begin{aligned} tw(G_\boxtimes )-1 \le tw(G_\boxslash ) \le tw(G) \le \max (4, tw\left( G_\boxtimes )\right) \end{aligned}$$

#### Proof

To start with, by noticing that the 4 extremities of the helix form a separator *S* between the inside and the outside of it, we get by Proposition 2 that $tw(G)\le \max (H\cup \text {\texttt{clique}}(S), G_\boxtimes )$. The graph $H\cup \texttt{clique}(S)$ does not depend on *G*, and consists of a helix with the 4 extremities forming a clique. With $l\ge 2$, it turns out that this graph has treewidth 4, per Lemma 1, hence the inequality.

Next, we notice that $G_\boxslash$ is a minor of *G* when $l\ge 3$. This can be seen by contracting the helix according to the pattern outlined on Fig. 3d by the green areas (each green area is contracted to the extremity it contains). Therefore, $tw(G_\boxslash )\le tw(G)$.

Finally, let us note that $G_\boxtimes$ and $G_\boxslash$ only differ by 1 edge, and removing a single edge from a graph can only decrease its treewidth by at most 1. Indeed, suppose that $tw(G_\boxslash )<tw(G_\boxtimes )-1$, and consider an optimal tree decomposition ${\mathcal {T}}$ for $G_\boxslash$. Let us denote by *u* and *v* the two extremities of the helix not connected in $G_\boxslash$. If the subtrees of bags containing respectively *u* and *v* do not intersect, then one can just add *v* to all bags of the tree decomposition, to represent the edge (*u*, *v*) while increasing the width by $\le 1$. Therefore $tw(G_\boxtimes )-1\le tw(G_\boxslash )$ and the inequality is complete. $\square$

Through the introduction of $G_\boxtimes$ and $G_\boxslash$ as the two possible graphs to which *G* is equivalent in terms of treewidth, Lemma 2 already contains the essence of our main structural result, Theorem 2. It will be the basis for generalizing tree decompositions of minimal expansions of a fatgraph to arbitrary helix lengths.

#### Theorem 2

If *H* is a helix in *G* of length $l\ge 5$, then extending the helix to have length $l+1$ does not increase the treewidth.

#### Proof

Let us distinguish two cases depending on the treewidth of *G*. For both of them, we consider an optimal tree decomposition ${\mathcal {T}}$ of *G* and show how to modify it into a valid tree decomposition for the extended version of *G*:

If $tw(G)\le 3$ then there has to be a pair *i*, *j* ($i\le j$) of indices $\in [1,l]$ such that $|i-j|>1$ and no bag contains both an element from$\{u_i,v_i\}$ and $\{u_j,v_j\}$. I.e. the occurences of $\{u_i,v_i\}$ and $\{u_j,v_j\}$ in the tree decomposition are completely separated by some edge (*X*, *Y*) of the tree decomposition. Indeed, if $\forall i,j\in [1,l]$ there is some edge between $\{u_i,v_i\}$ and $\{u_j,v_j\}$ represented, then contracting $u_k,v_k$ together $\forall k$ would yield a clique on 5 vertices, which is forbidden if $tw(G)\le 3$.

Given such a pair *i*, *j* of indices, let us denote $S=X\cap Y$ the separator associated to that edge. By Proposition 3, *S* can be assumed to be inclusion minimal, and therefore to contain exactly 2 vertices $u_k$ and $v_{k'}$ such that $|k-k'|\le 1$ and $i\le k,k'\le j$. Such a separator is depicted on Fig. 3c, as well as on Fig. 7. On this latter Figure, we also depict the re-writing we perform: we introduce two new vertices *x* and *y* to the *X*-side of the separator, as well as intermediary bags between *Y* and *X* that will gradually transform $u_k,v_k'$ into *x* and *y*. To be specific, we introduce *S* as a bag between *X* and *Y*, and connect it to *X* through the series of bags $S\cup \{x\}$, $S\cup \{x,y\}\setminus \{u_k\}$, $S\cup \{x,y\}{\setminus } \{u_k,v_k'\}$ in the case (w.l.o.g) that $k\le k'$. In addition, all occurences of $u_k$ in *X* and beyond in the subtree rooted at *X* and directed away from *S* are replaced with *x* and those of $v_k'$ with *y*. Since $|S|\le tw(G)$, such a re-writing does not increase the treewidth, while representing all necessary edges for an extension of the helix by one level.

If $tw(G) \ge 4$, then we first look for a pair *i*, *j* verifying (as above) that some edge (*X*, *Y*) of the tree decomposition completely separates $\{u_i,v_i\}$ from $\{u_j,v_j\}$, although this time with no garantee of finding one. If we do find one, we apply the same transformation as above.

In the case where no such pair *i*, *j* exists, we argue that the four extremities of the helix form a safe separator of *G*. i.e. $tw(G)=\max (4,tw(G_\boxtimes ))$. An optimal tree decomposition for *G* can then be obtained from a tree decomposition $G_\boxtimes$, and a tree decomposition of an helix closed by a clique, connected through a bag in which the separator forms a clique. The helix can then simply be extended by changing the part of the tree decomposition representing the helix.

By Lemma 2, we have $tw(G)\le \max (4,tw(G_\boxtimes ))$. Since $tw(G)\ge 4$, it reduces to $tw(G)\le tw(G_\boxtimes )$. We now use the fact that edges connecting $\{u_i,v_i\}$ and $\{u_j,v_j\}$ for all *i*, *j* are represented in the tree decomposition to show that $G_\boxtimes$ is a minor of *G*, and therefore $tw(G)=tw(G_\boxtimes )$

If there is an edge connecting $u_i$ to $v_j$ or $v_i$ to $u_j$ for $|j-i|>1$ represented in the tree decomposition, then we obtain $G_\boxtimes$ through the contraction scheme represented on Fig. 3. If $\forall i,j$ the edge connecting $\{u_i,v_i\}$ and $\{u_j,v_j\}$ is $(u_i, u_j)$ or $(v_i,v_j)$, then w.l.o.g we are in one of the two situations colored in orange on Fig. 3. By contracting the orange parts into the extremity they contain, we get $G_\boxtimes$ as a minor of *G*. $\square$

Since bulges in a helix only consist of vertices of degree exactly 2, combining Proposition 1 with Theorem 2 implies that the treewidth of any expansion of a given fatgraph is always smaller than or equal to the treewidth of a minimal expansion where all bands are helices of length exactly 5. As for gaps, arguments similar to the proof of Theorem 2 can show that going from a gap of length 0 to an arbitrary length does not increase the treewidth of a fatgraph expansion. Overall, we formally define the minimal expansion of a fatgraph as:

#### Definition 4

(Minimal representative expansion of a fatgraph) Given a fatgraph $\gamma$, its minimal representative expansion consists of:

- A perfect helix of length 5 for each band.
- No gap between the extremities of two helices

Such a minimal representative expansion is illustrated in Fig. 8a. For visual clarity, gaps have been kept between consecutive helices, but one can see that the corresponding extremities have the same labels. Given a fatgraph, this RNA structure graph contains all necessary information for formulating DP equations decomposing all RNA structures compatible with the fatgraph.

Interestingly, the two graphs $G_\boxtimes$ and $G_\boxslash$ that emerge in the proofs as the two graphs *G* could be equivalent in terms of treewidth, as well as the separators they are associated to (see Fig. 3c) are reminiscent of two typical decomposition strategies used into dynamic programming for RNA folding. They suggest, for each helix in a graph, two possible “canonical representations” in terms of tree decomposition, which will be elaborated on in the next section.

## Interpreting the tree decomposition of a fatgraph expansion as a DP algorithm

Starting with a tree decomposition for a minimal representative expansion of a given fatgraph, we first describe in this section how to represent it in a *canonical form*, with each helix represented either in one of two different ways, respectively related to $G_\boxslash$ and $G_\boxtimes$. The resulting tree decomposition can be further compressed into a *skeleton*, where bags within individual helices are compressed into a single bag.

This tree can then be interpreted as a dynamic programming scheme, in which helices are generated by specializing dynamic programming subroutines. In a sense, the tree decomposition yields automatically a decomposition strategy usable for dynamic programming, of the kind that was hand-crafted in previous approaches [11, 12].

### Canonical form of fatgraph tree decompositions

Let us recall this additional definition for the sake of presentation: Given an edge $e=(X,Y)$ of a tree decomposition ${\mathcal {T}}$, we call the $X-side$ of ${\mathcal {T}}$ the connected component of $T\setminus e$ containing *X*.

#### Definition 5

A tree decomposition of an expansion *G* of a fatgraph is in canonical form if, for each helix *H* of length *l*, either:

- **Clique case:** *H* is represented by a root bag that contains its 4 extremities, connected to a sub-tree-decomposition $T_l$ recursively defined as
  $$\begin{aligned} T_0^{\boxtimes }&=\emptyset \\ T_l^{\boxtimes }&=\{u_1,v_1,u_l,v_l\}\\&\rightarrow \{u_1,v_1,u_l,v_{l-1},v_l\}\\&\rightarrow \{u_1,v_1,u_{l-1},u_l,v_{l-1}\}\rightarrow T_{l-1}^{\boxtimes } \end{aligned}$$
- **Diagonal case:** Helix *H* is represented by a linear series of bags starting with $X_1=S^{*}\cup \{u_1,v_1\}$, finishing with $X_{2l+2}=S^{*}\cup \{u_l,v_l\}$, and such that for $1<k<l+1$:
  $$\begin{aligned} X_{2k}=S^{*}\cup \{u_{2k-1},v_{2k-1},u_{2k}\} \end{aligned}$$
  and
  $$\begin{aligned} X_{2k+1}=S^{*}\cup \{v_{2k-1},u_{2k},v_{2k}\}. \end{aligned}$$

**Fig. 4:**
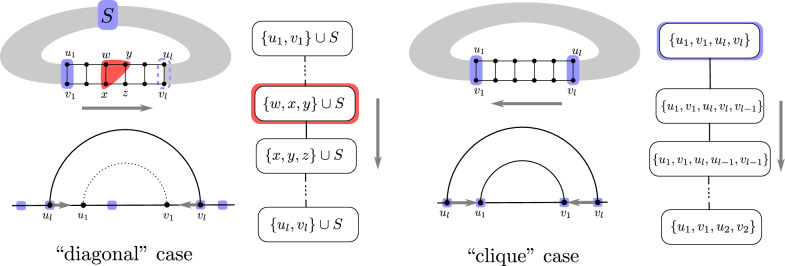
The two types of canonical representations for the helices of a graph completion *G* and associated dynamic programming schemes. (Left) In the Diagonal case, only the sequence positions of external (resp. internal) anchors are provided. Internal ones are obtained as the base case of an energy model-dependent dedicated dynamic programming scheme, propagating values for anchors in *S* along the way. (Right) In the clique case, all four anchors delimiting the helix have known position. Again, a dedicated dynamic programming algorithm is used to optimize over all possible contents for the helix, while accounting for associated free-energies

**Fig. 5:**
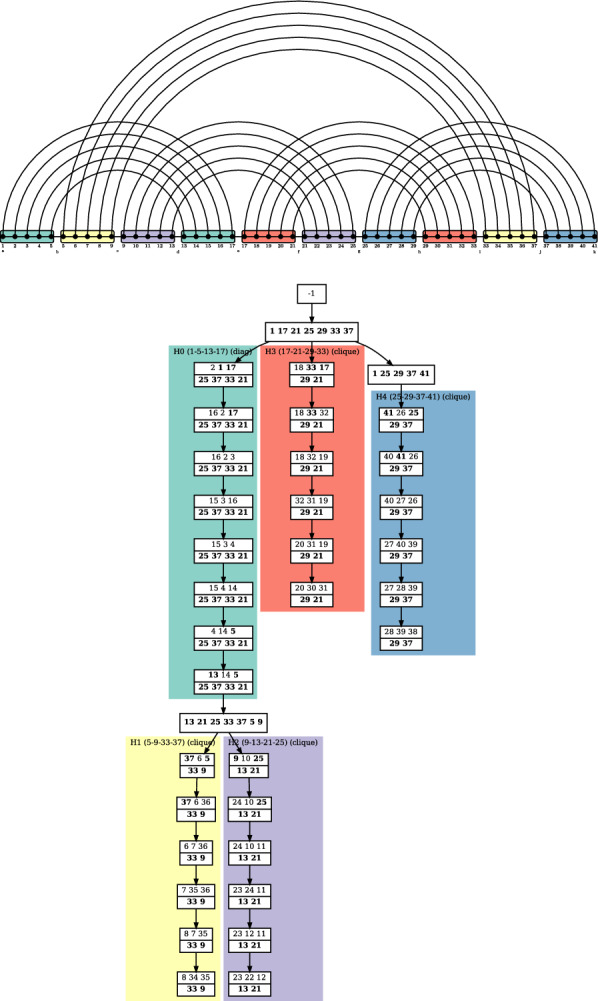
Canonical tree decomposition of the fatgraph given in Fig. 1. White boxes represent the bags of the tree decomposition. Number in the bags correspond to the indices of the helices in the fatgraph where number on the bottom are kept while traversing the branch of the decomposition tree. Colored frames indicate the distinct helices (H0 to H4) of the structure. The tree decomposition was computed with the optimal solver [31], which we noticed is particularly efficient on RNA structure graphs

**Fig. 6:**
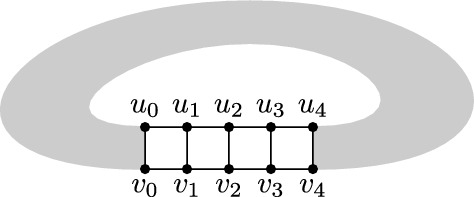
Sketch of an helix subgraph, in a minimal representative expansion of a fatgraph, along the annotation of vertices used in Algorithm 2. There is a slight abuse in using these same labels for each of the helices in the main for loop of Algorithm 2

The definition above is illustrated by Fig. 4. A canonical tree decomposition for a minimum expansion of a fatgraph is also presented on Fig. 5. It was obtained through the processing routine that we describe in Algorithm 2, applicable to any (optimal or not) tree decomposition. It can therefore use a sub-optimal tree decomposition obtained from a polynomial heuristic [21] instead of an exponential solver, if the latter is too time-consuming (although [31] is empirically quite efficient on RNA structure graphs) (Fig 6).

Algorithm 2 essentially follows the dichotomy of the proof of Theorem 2. We state its correctness, run-time and proof below.

**Figure:**
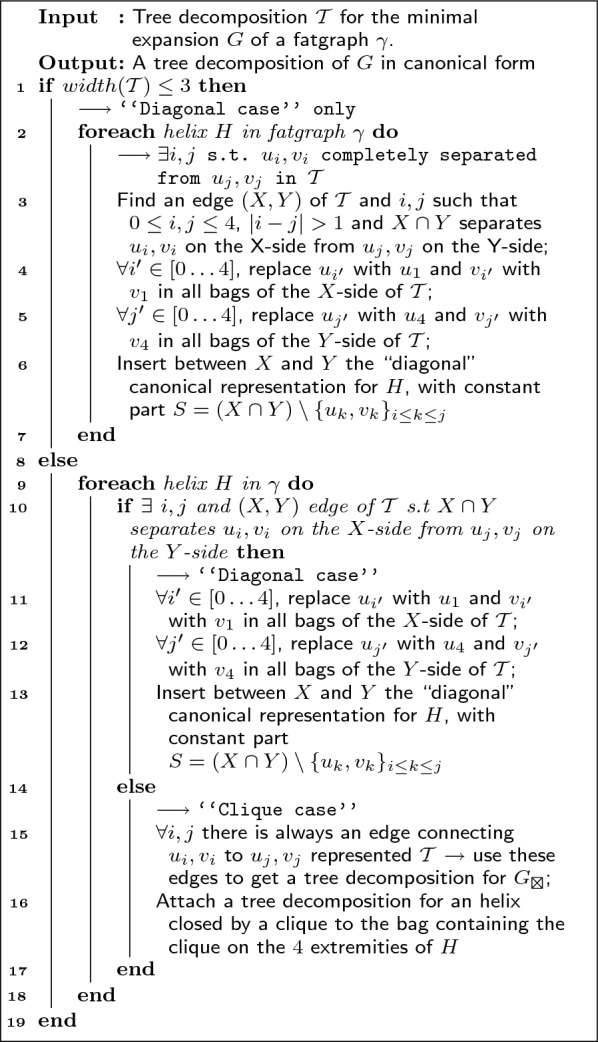
**Algorithm 2:** Algorithm for re-writing a tree decomposition into a canonical one in which every helix of the input graph is represented in a canonical way. A representation of an helix as a subgraph in a minimal representative expansion, along with the notations (ui, vj ...) used in this pseudo-code can be found on Figure 6. With a slight abuse of notation, we re-use these variables for each helix.

#### Theorem 3

Given *G* the structure graph of a minimal expansion of a fatgraph $\gamma$, and ${\mathcal {T}}$ a tree decomposition of *G*, Algorithm 2 outputs a canonical tree decomposition for *G*, having same width as *T*, in time $O(N_H\cdot n^3)$, where $N_H$ is the number of helices in $\gamma$.

#### Proof

Concerning the run-time, enumerating all pairs $1\le i<j\le l)$ is quadratic in the length of the helix under consideration, which is *O*(*n*) in a general graph, while testing a given edge for separation of $u_i,v_i$ and $u_j,v_j$ takes *O*(*n*) (through breadth-first search) for each of the *O*(*n*) edges of the tree decomposition.

**Fig. 7:**
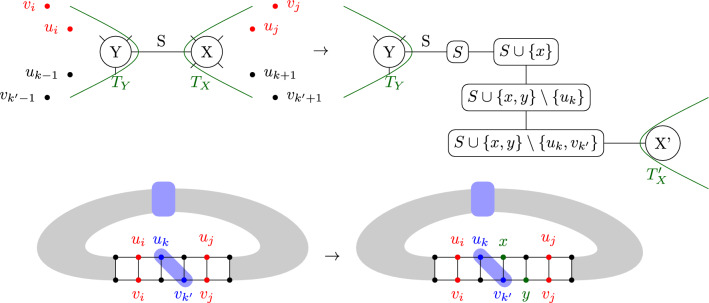
Representation of the local rewriting of a tree decomposition next to a separator *S* separating to base pairs $(u_i,v_i)$ and $(u_j,v_j)$, in order to extend a helix by one unit, through the introduction of new vertices *x* and *y*. This is used in Theorem 2, in what corresponds in Sect "Interpreting the tree decomposition of a fatgraph expansion as a DP algorithm"  to the “diagonal” case

As for its correctness: it essentially follows the dichotomy of Theorem 2. If $width({\mathcal {T}})\le 3$, then there has to be a pair of indices *i*, *j* such that $\{u_i,v_i\}$ is separated from $\{u_j,v_j\}$ by an edge (*X*, *Y*) of the tree decomposition. If it is not the case, contracting $(u_k,v_k)$ $\forall k$ yields a $K_5$-minor, which is not possible with a width of 3. We therefore get a separator as depicted in blue on Fig. 7, which forms the “constant part” of the diagonal-case helix representation. The replacement of vertex occurences on both sides of the separator does not increase the width, while representing all edges of the graph.

If $width({\mathcal {T}})\ge 4$, if a separator as above is found (but this time, no guarantee to find one), then we apply the same transformation. Otherwise, we use the extra edges represented in the tree decomposition to modify it into a tree decomposition of $G_\boxtimes$, as in the proof of Theorem 2. There is then necessarily a bag containing all four extremities of the helix, to which a tree decomposition representing the inside of the helix can be attached. $\square$

Note that in a canonical tree decomposition, all vertices and edges internal to a helix of a graph are represented in the canonical sub-tree-decomposition associated to it. All bags outside of these canonical blocks only consist of extremities of helices, or other vertices outside of helices. Ignoring these internal parts, to focus on a more compact “skeleton” of canonical tree decompositions will be the first step towards automatically deriving dynamic programming equations.

#### Definition 6

The skeleton of a canonical tree decomposition for a graph *G*, is defined as follows:

- All sub-tree-decompositions representing a helix in the “clique” case are replaced with a unique bag containing all extremities of the helix
- All sub-tree-decompositions representing a helix in the “diagonal” case are contracted to contain their first and last bags only, denoted as $S\cup \{u_1,v_1\}$ and $S\cup \{u_l,v_l\}$ in Definition 5.

**Fig. 8:**
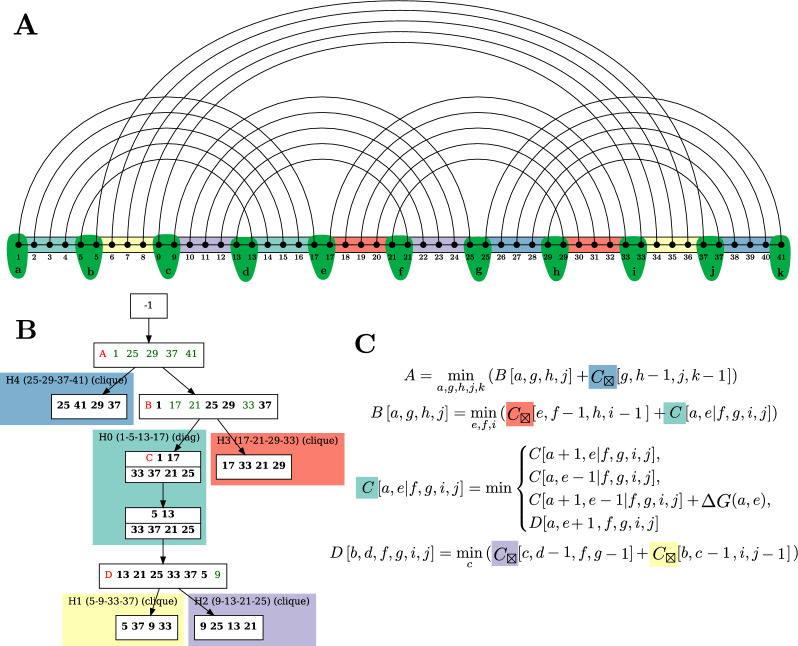
**A** Minimal representative length-5 expansion of the fatgraph shown in Fig. 1. Anchor variables are highlighted in green. We introduce one such variable per gap between helices. **B** Skeleton of the tree decomposition. White boxes represent transitional bags, introducing/propagating indices, while colored boxes represent helices in the fatgraph (H0 to H4) with associated indices in the input structure. Red letters indicate tables of the dynamic programming algorithm. Green indices are novel indices, absent from a bag’s predecessor. **D** P equations derived from the compact skeleton, involving the anchor variable defined above, and following the rules described in Sect. "Automatic derivation of dynamic programming equations in a base pair-based energy model"

Figure 8b gives an example of such a skeleton.

### Automatic derivation of dynamic programming equations in a base pair-based energy model

Given the skeleton of a representative minimal expansion of a fatgraph $\gamma$, we describe here how to formulate DP equations for the corresponding folding problem. We initially restrict our exposition to a base-pair based model, further named BP model, akin to the one optimized by the seminal Nussinov algorithm [38], where the free-energy of a structure *S* is given by:

$$\begin{aligned} E_{BP}(S) = \sum _{(i,j)\in S} \Delta G_{i,j}, \end{aligned}$$

$\Delta G_{i,j}$ being the contribution of a base-pair (*i*, *j*) to the free-energy (or negative log-odd to produce max-likelihood structures).

Essentially, we introduce helix DP tables for each helix, and transitional tables for non-helix bags. The variables indexing these tables are called *anchors*. These integer variables each represent a separation point between consecutive (half-)helices. Taken together, a full set of anchors $(a,b,c,\ldots )$ partitions the sequence into a set of disjoint intervals $[a,b[,[b,c[\ldots$, each associated with one *half-helix*, i.e. one of the subsequences that form a helix. Helix tables will account for the free-energy contributions of concrete base-pairs, while transitional tables will instantiate anchors in a way that remains consistent with previous assignments.

Indeed, owing to the definition of a valid tree decomposition, a skeleton is guaranteed to:

1. Feature each anchor in some bag;
2. Represent each pair of consecutive anchors in at least one bag;
3. Propagate anchor values, such that the anchor values within helix tables remain consistent. This implies that non-helix bags can simply propagate previously-assigned anchors, possibly assigning values to novel anchors (if any and constrained to remain consistent with the sequential order) to explore all possible partitions of the input RNA sequence.

Helix tables will predict concrete sets of base pairs and account for their associated free-energy. In order to both prevent the double pairing of certain sequence positions, and to avoid ambiguity, we require (and enforce in the DP rules) that an anchor *x*, separating the consecutive halves of two helices *H* and $H'$, implies the pairing of position *x* to the other half of $H'$, and the pairing of some position $x'<x$ as part of *H*. In other words, a helix *H* delimited by anchors $i,i',j',$ and *j* must pair position *i* to some position $x\in ]j',j[$, and $j'$ to some position $y\in ]i,i'[$, implicitly leaving both regions $]y,i'[$ and ]*x*, *j*[ unpaired.

#### Helix table 1: “Clique” cases

In the skeleton, each bag representing a helix in the “clique” case is associated to the following tables, where $i, i'+1, j'$, and $j+1$ represent the values of the anchors delimiting the helix. The increments on $i'$ and *j* are here to ensure the presence of gap of length $\ge 1$ between two base pairs belonging to different helices. (see also Fig. 8c for an example of how anchor values are passed to $C_\boxtimes$ with a decrement of $-1$ for the same reason).

A first table $C_\boxtimes '$ holds the minimal free-energy of a helix delimited by $i,i',j',$ and *j*, such that position *i* is paired to some $x\in ]j',j[$ and $j'$ to some position $y\in ]i,i'[$. The idea is here to iteratively move the anchor from *j* to $j-1$, implicitly leaving position *j* unpaired, until a base pair (*i*, *j*) is formed. Once a base pair is created, we transition to another table $C_\boxtimes$ which optimizes over helices like $C_\boxtimes '$, but additionally allows position *i* to be left unpaired.

Those two tables can be filled owing to the following recurrences:

$$\begin{aligned} C_\boxtimes '[i,i',j',j]=\min {\left\{ \begin{array}{ll} C_\boxtimes '[i,i',j',j-1] \quad {\{\text {if }j'<j\}}\\ C_\boxtimes [i+1,i',j',j-1] + \Delta G_{i,j}\\ \quad {\{\text {if }(i<i')\wedge (j'<j)\}}\\ \Delta G_{i,j} \quad {\{\text {if }j=j'\}}\\ +\infty \quad {\{\text {if no case applies}\}} \end{array}\right. } \end{aligned}$$

and

$$\begin{aligned} C_\boxtimes [i,i',j',j]=\min {\left\{ \begin{array}{ll} C_\boxtimes '[i,i',j',j-1] \quad {\{\text {if }j'<j\}}\\ C_\boxtimes [i+1,i',j',j] \quad {\{\text {if }i<i'\}}\\ C_\boxtimes [i+1,i',j',j-1] + \Delta G_{i,j}\\ \quad {\{\text {if }(i<i')\wedge (j'<j)\}}\\ \Delta G_{i,j} \quad {\{\text {if } j=j'\}}\\ +\infty \quad {\{\text {if no case applies}\}} \end{array}\right. } \end{aligned}$$

where $\Delta G_{i,j}$ denote the free-energy contribution of the base pair (*i*, *j*) in the input RNA sequence.

#### Helix tables 2: “Diagonal” cases

In the skeleton bags representing the diagonal cases, we need to associate a different table to each helix. Indeed, each “diagonal” case associates, to a helix *H*, a set *S* of indices, dubbed the *constant anchors*, whose values remain unchanged during the construction of *H*.

**Fig. 9:**
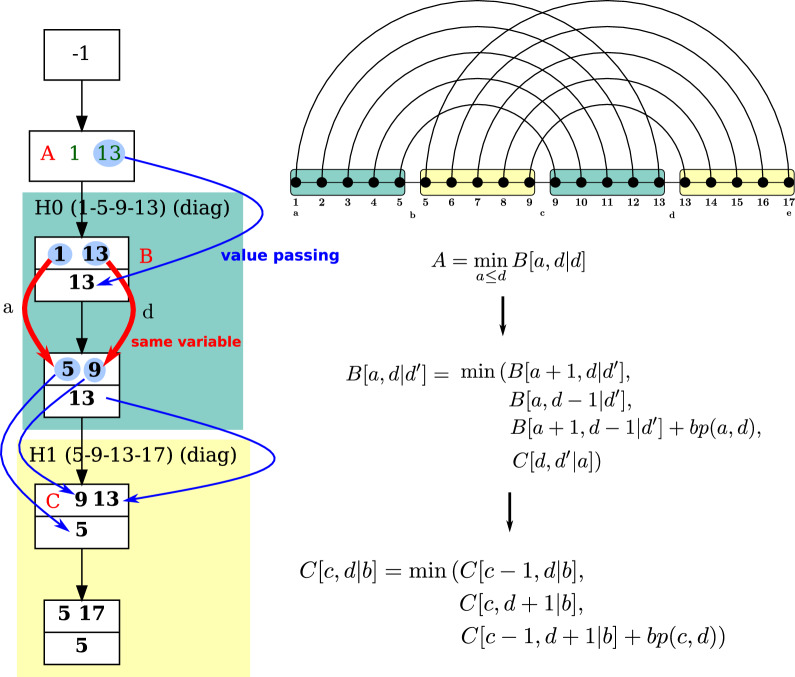
Derivation of DP equations from a skeleton, starting from the canonical tree decomposition of a length-5 expansion for a simple *H*-type fatgraph. On the left-hand-side, special emphasis is given to explaining how values are mapped at the end of a diagonal case. Extra tables $C_\boxtimes '$ and $D'_H$, needed to ensure unambiguity of the DP scheme, are omitted for the sake of simplicity without adverse consequences to correctness

We focus on the case where (*i*, *j*) represents the value of the outermost anchor pair (i.e. [*i*, *j*] represents the full span of *H*), leaving to the reader the symmetric case starting from the innermost pair. Note that, in the skeleton, we kept two bags for a “diagonal case” helix. Yet they are associated to a single table, since the helix is created by incrementing two indices only, such that the initial pair of extremities “becomes” the other pair. We need this second bag to know how to map index values to the children tables $\{M_k\}_k$. This value mapping at the end of a diagonal case is illustrated on Fig. 9.

Namely, let the cell $D_H[i,j\mid S]$ (resp. $D'_H[i,j\mid S]$) represent the minimum-free energy achieved by the set of helices in the subtree of *H*, when *H* is anchored at (*i*, *j*) without commitment to form base pairs for neither *i* nor *j* (resp. where *i* is committed to form a pair with some position $x\le j'$). We have:

$$\begin{aligned} D_H'[i,j \mid S] = \min {\left\{ \begin{array}{ll} D_H'[i,j-1\mid S] \\ \quad {\{\text {if }j-1>i\wedge \forall s\in S,\;j-1\ne s\}}\\ D_H[i+1,j-1\mid S]+\Delta G_{i,j}\\ \quad {\{\text {if }\forall s\in S,\;(i+1 \ne s)\wedge (j-1\ne s)\}} \end{array}\right. } \end{aligned}$$

and

$$\begin{aligned} D_H[i,j \mid S] = \min {\left\{ \begin{array}{ll} D_H[i+1,j\mid S] \\ \quad {\{\text {if }i+1<j \wedge \forall s\in S,\;i+1\ne s\}}\\ D_H'[i,j-1\mid S] \\ \quad {\{\text {if }j-1>i\wedge \forall s\in S,\;j-1\ne s\}}\\ D_H[i+1,j-1\mid S]+\Delta G_{i,j} \\ \quad {\{\text {if }\forall s\in S,\;(i+1 \ne s)\wedge (j-1\ne s)\}}\\ \sum _{k} M_k[I_k]\\ \quad {\{\text {with }I_k:= \left( \{i,j+1\}\cup S\right) \cap A_k\}} \end{array}\right. } \end{aligned}$$

where $A_k$ denotes the anchors values needed for the *k*-th child of the diagonal bag.

#### Transitional tables: Non-helix bags

The general case consists of passing the values of relevant variables onward to the diagonal and clique tables, possibly assigning/propagating anchors that appear in the bag for the first time, i.e. anchors that are not found in the parent bag. Let $I_P$ be the anchors of the parent bag of *M* in the tree decomposition, we have:

$$\begin{aligned} M[I_P]=\min _{\begin{array}{c} \text {Values for}\\ \text {anch. in } I\setminus I_P \end{array}} \sum _{k=1}^{\#\text {child.}} {\left\{ \begin{array}{ll} M_k[I_k] \\ \quad {\{\text {if { k}-th child trans.}\}}\\ C_\boxtimes '[i,i'-1,j',j-1] \\ \quad {\{\text {if clique at }(i,i',j',j)\}}\\ D'_{H_k}[i,j-1\mid S_k] \\ \quad {\{\text {if diagonal at }(i,j')\}}\\ \end{array}\right. } \end{aligned}$$

where $I_k$ denotes the anchor values from *I* needed for the *k*-th child of the bag, and *S* represents the constant anchors of the *k*-th child, assumed to be a diagonal.

#### Complexity analysis

Let $w_{\boxtimes }$, $w_\boxslash$ and $w'$ be the maximum width of a clique, diagonal and transitional bag (i.e. its size minus one; or 0 if no bag exist for a given type) in a canonical tree decomposition ${\mathcal {T}}$ of a fatgraph $\gamma$. Note that $w_\boxtimes$ is always 4, but we keep this notation for consistency. In the following theorem, $\gamma$ is a fatgraph with $|\gamma |$ helices and ${\mathcal {T}}$ is a canonical tree decomposition for $\gamma$. The DP scheme obtained from ${\mathcal {T}}$ as described in the previous section is called the DP scheme *inferred from* ${\mathcal {T}}$.

##### Theorem 4

In the base-pair energy model, the DP scheme inferred from ${\mathcal {T}}$ yields an algorithm for the Fatgraph MFE Folding problem with $O(|\gamma |\cdot n^{max(w_{\boxtimes },w_\boxslash ,w'+1)})$ time and $O(|\gamma |\cdot n^{max(w_{\boxtimes },w_\boxslash ,w')})$ space complexity.

##### Proof

The complexity of the DP scheme inferred from ${\mathcal {T}}$ (presented in the previous section for a base-pair based model) depends on the complexities of filling each of the tables corresponding to helices.

$C_\boxtimes [i,i',j',j]$ and $C_\boxtimes '[i,i',j',j]$ take $O(n^4)$ to fill, using either a memoization procedure or a bottom-up iteration of all possible values for $i,i',j',j$. It is equal to the space complexity thanks to the finite number of cases in their recursive equations.

A similar analysis holds for $C_\boxslash [i,j\mid S]$ and $C_\boxslash '[i,j\mid S]$, except that the number of indices is $|S|+2$. Since the maximum size of a bag in a diagonal-case representation is $|S|+3$, we indeed have $w_\boxslash =|S|+2$.

For transitional bags, the situation is slightly different. The indices of the table are the intersection with the parent bag in the tree decomposition, whose number is bounded by $w'$. The space complexity of the corresponding DP table is therefore $O(n^{tw'})$. But there is also a minimization over all possible values for the variables not present in the parent bag, incurring a linear factor for each of them. Overall, for a transitional *B* of maximum size $w'+1$, the complexity of filling the matrix is $O(w'+1)$ ($O(n^{|B\setminus P|})$ for each of the $O(n^{|B\cap P|})$) entries.

As for the number of tables, it is at most twice the number of bags in ${\mathcal {T}}$, which is linear in the number of helices in $\gamma$. The overall time complexity is therefore given the DP table of most expensive filling cost, $O(|\gamma |\cdot n^{\max (w_\boxtimes ,w_\boxslash ,w'+1)})$. The same holds for the space complexity, yielding $O(|\gamma |\cdot n^{\max (w_\boxtimes ,w_\boxslash ,w')})$. $\square$

Since tree decompositions are typically chosen to minimize their width $tw:=max(w_{\boxtimes },w_\boxslash ,w')$, then the precise resulting complexity may depend on the choice of an optimal tree decomposition. Indeed, it could be that $tw = w'$, yielding a $O(n^{tw+1})$ algorithm or, conversely, $w'< tw-1$ would imply a complexity of $O(n^{tw})$. In other words, in the base pair model, the algorithm induced by the choice of an arbitrary tree decomposition *T* may be suboptimal by a linear factor. Figure 11 shows an example with two tree decompositions of the same width, but with different $w'$ values. They yield different complexities ($O(n^4)$ vs. $O(n^5)$).

Fortunately, it is possible to work around this issue, and obtain a $O(n^{tw})$ DP algorithm anytime a suitable canonical fatgraph decomposition exists. To find such a decomposition, we explore the space of all possible canonical tree decompositions, through an enumeration of all possible representations for each helix. This is formalized in the theorem below (note that this is purely meant as a *feasibility* result, we do not expect this approach to be optimal in terms of complexity; however, we conjecture that this subproblem is FPT for the treewdidth of $\gamma$). We use the same notations as above by calling $w'({\mathcal {T}})$ the maximum width of a transitional bag of a canonical tree decomposition.

##### Theorem 5

Let *G* be a minimal expansion of a fatgraph $\gamma$ with $n_H$ helices. If there exists an optimal canonical tree decomposition ${\mathcal {T}}$ of *G* such that $w'({\mathcal {T}})\le tw(G)-1$, then such a ${\mathcal {T}}$ can be found in $2^{O\left( |\gamma |^2\right) }\cdot f(tw)$ time.

##### Proof

The space of all possible canonical tree decomposition can be iterated over by deciding, for each helix, whether it is in the “clique” or “diagonal” case. If it is in the diagonal case, one must in addition decide what is the “constant part” of the representation of the helix. Any set *S* such that $\{u_1,v_1,u_5,v_5\}\cup S$ separates the graph into at least 3 connected components, one being the inside of the helix, is an eligible candidate.

This process corresponds to deciding, for each helix, what separator cuts out the inside of the helix from the rest of the graph. When such a decision is made, a canonical tree decomposition can be obtained by computing canonical tree decompositions for the connected components associate to the separator, and connecting them together (in the spirit of Proposition 2).

When there are no helices left, an optimal tree decomposition of the graph is computed in time *f*(*tw*). It yields the transitional bags in between helix representations.

Given that *S* is only composed of helix extremities, it is chosen among $\le |\gamma |$ vertices. We consider therefore an upper bound of $2^{|\gamma |}$ for the number of possible choices of *S* in the diagonal case, and an upper bound of $|\gamma |$ for the number of connected components associated to a separator, the overall time of exploring all canonical tree decompositions is bounded by $O((|\gamma |\cdot 2^{|\gamma |})^{|\gamma |}\cdot f(tw)) \subseteq 2^{O\left( |\gamma |^2\right) }\cdot f(tw)$.

If an optimal canonical tree decomposition ${\mathcal {T}}$ such that $w'({\mathcal {T}})\le tw(G)-1$ exists, then it corresponds to a particular assignation of separators to each helix as outlined above, and it will be one of the tree decompositions explored by the iteration. $\square$

### Automated C code generation

**Fig. 10:**
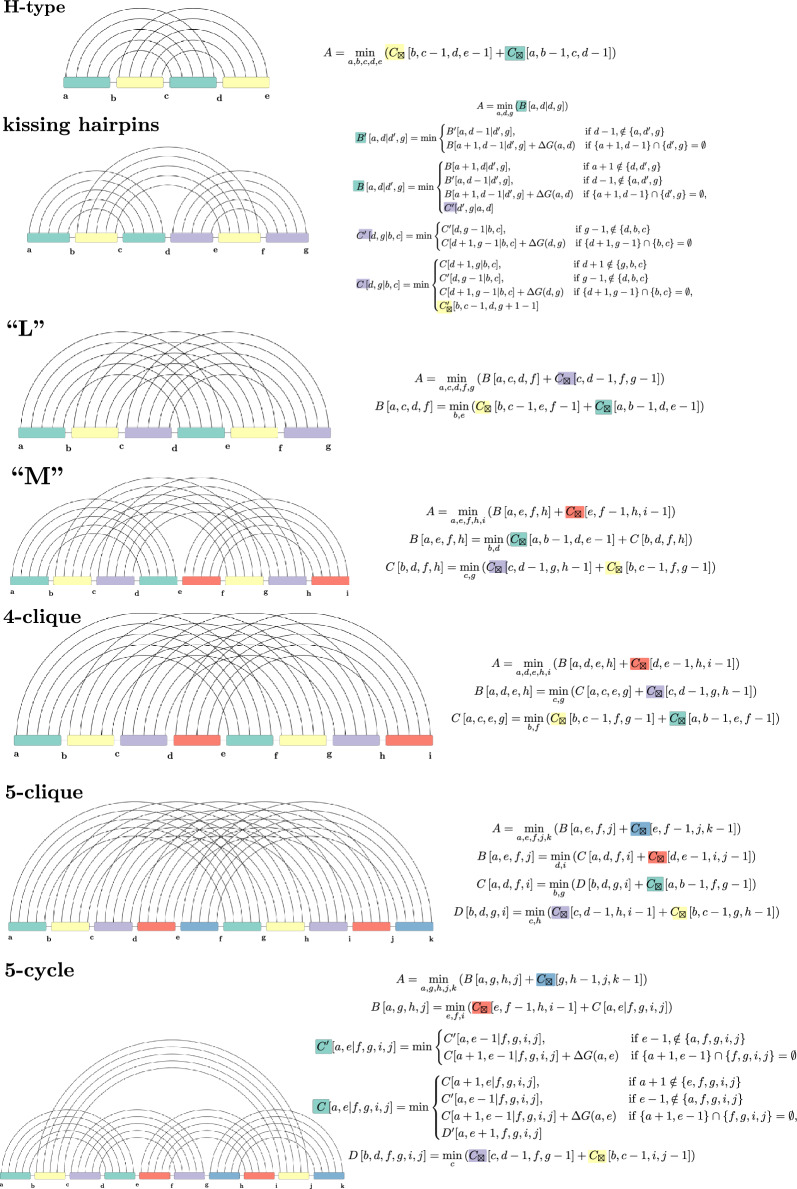
Minimal representative expansions and final equations for the examples of Table 2. The equations have been automatically generated, and the pipeline code is freely available at https://gitlab.inria.fr/bmarchan/auto-dp. In particular, the optimal tree decompositions were computed using an exact algorithm proposed by Tamaki [31]

**Fig. 11:**
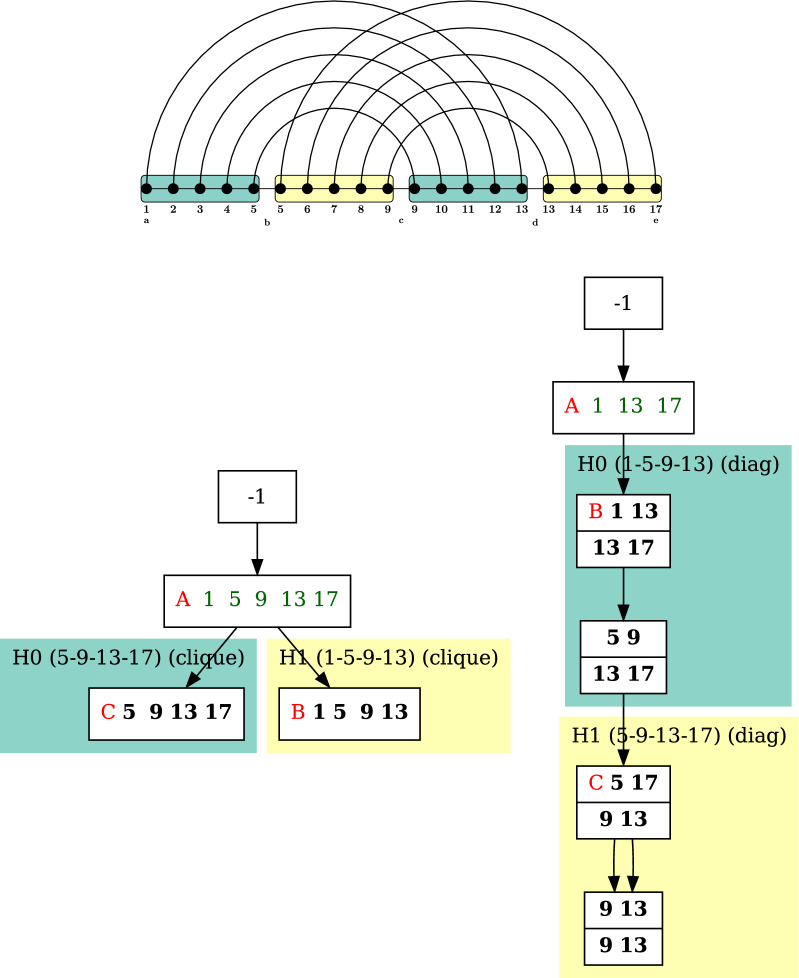
Example of two tree decompositions of the same width, yielding different complexities. The decomposition on the left has $w_\boxslash =0$, $w_\boxtimes =4$ and $w'=4$, which gives a complexity of $O(n^5)$ (Theorem 4). On the other hand, the decomposition on the right has $w_\boxslash =4$, $w_\boxtimes =0$ and $w'=3$, which yields $O(n^4)$

Figure 8 shows an example of output to our pipeline, with automatically generated LaTeX equations for the dynamic programming scheme inferred from the tree decomposition. Figure 10 gives other examples of such automatically generated equations. But our implementation, available freely at https://gitlab.inria.fr/bmarchan/auto-dp, is also capable of automatically generating C code implementing these equations. The automatically generated *.c files corresponding to all of the examples of Fig. 10 are available as Supplementary Material. In the current state, they are only meant as a prototype demonstration. Developments towards generation of fully functional code, including the extensions presented in the next Section, will be the subject of future work.

## Extensions

The DP scheme, as stated above, only supports conformations that consist of a single pseudoknot configuration, indicated by a fatgraph. Moreover, it forces the first position of the sequence to always form a base pair. Finally, it considers an energy model that is fairly unrealistic in comparison with the current state of the art. In this section, we briefly describe how to extend this fundamental construction in several directions. This enables us to solve the stated algorithm design problem (Def. 2) and consequently the associated folding problem in complex energy models, and discuss the consequences on the complexity.

### Integration with classic DP algorithms for MFE structure prediction

Firstly, let us note that alternative fatgraphs can easily be considered, without significant overhead, by adding a disjunctive rule at the top level of the DP scheme, such as

$$\begin{aligned} \text {MFE}_{\text {PK}}:= \min _{i=1}^p \text {root}_{\gamma _i} [\emptyset ] \end{aligned}$$

where $\text {root}_{\gamma _i}$ is the top level case of the DP scheme for fatgraph $\gamma _i$.The associated conformation space then consists of the union of all pseudoknotted structures compatible with one of the fatgraphs.

#### Enriching classic schemes with fatgraphs

Fatgraphs usually represent a structural module rather than a complete RNA conformation. The classic DP scheme for 2D structure energy-minimization can thus be supplemented by additional constructs, enabling the consideration of pseudoknots. Towards that goal, one needs to access $\text {MFE}_{\text {PK}}(i,j)$, the MFE achieved over a region [*i*, *j*] by a conformation compatible with one of the input fatgraphs. In other words, one needs to be able to prescribe the span of the fatgraph occurrence, i.e. the values (*i*, *j*) of its extremal anchors $(a,a')$ within the dynamic programming.

To ensure this possibility, one simply needs to connect the first and last positions within the minimal fatgraph completion $G=(V,E)$, i.e. resulting in a graph $G':=(V,E\cup \{(a,a')\})$. Since each arc of the input graph is represented in a valid tree decomposition, we know that any tree decomposition for $G'$ features a bag *B* including both *a* and $a'$, possibly in conjunction with additional anchors $S:=\{k_1,k_2,\ldots \}$. Moreover, since a tree decomposition is unordered, it can be rerooted to start with *B*, and preceded by a root node restricted to anchors (*a*, *b*), without adverse consequences complexity-wise. This yields the following entry point for the DP of a fatgraph $\gamma$:

$$\begin{aligned} \text {MFE}_{\gamma }(i,j):= \min _{i<k_1<k_2<\ldots <j}M_B[i,k_1,k_2,\ldots ,j] \end{aligned}$$

which can be used within a classic, pseudoknot-oblivious, DP scheme for MFE structure prediction. Complexity-wise, it can be shown that the additional base pair can at most increase by 1 the treewidth (and frequently leaves it unchanged).

#### Recursive substructures

Recursive substructures consist of secondary structures/occurrences of fatgraphs that are inserted, both in between and within helices, usually through recursive calls to the (augmented) 2D folding scheme.

To allow arbitrary sub-structures to be inserted in the gaps between consecutive helices, one can again modify the minimal helix expansion to distinguish the anchors *a*, *b* associated with consecutive helices (instead of merging them into a single anchor in our initial exposition). By connecting *a* and *b*, one ensures their simultaneous presence in a tagged bag *B*, whose DP recurrence is then augmented to include an energy contribution $\text {MFE}_{\text {SS}}(a+1,b-1)$.

To enable the insertion of substructures within a helix requires modifications to the helix clique/diagonal rules that are very similar to the ones enabling support for the Turner energy model. Assuming the presence of a base pair (*i*, *j*), an insertion can indeed be performed by delimiting a region [*i*, *k*] (resp. [*k*, *j*]) of arbitrary length, leading to an overall MFE of $\text {MFE}_{\text {SS}}(i,k) + \delta$, where $\delta$ is the free-energy contributed by the rest of the helix (e.g. to include additional terms associated with multiloops).

**Table 1:** While the space complexity of the generated DP schemes is always bounded by $O(n^{tw})$ (Lemma 3), the run-time complexity of filling-up the DP tables $C_{\boxslash }$ and $C_{\boxtimes }$ depends on the choice of energy model. As for the table corresponding to a transitional bag *X* with indices *I*, the cost of filling it is $O(n^{tw+1})$ irrespectively of the energy model

Energy model	Diagonal tables	Clique tables	Transitional tables
	$C_{\boxslash }[i,j\vert S]$	$C_{\boxtimes }[i,i',j',j]$	$M_X[I_X]$
BP-based model	$O\left( n^{\vert S\vert +2}\right)$	$O\left( n^4\right)$	$O\left( n^{|I|}\right)$
BP+stacking	$O\left( n^{|S|+2}\right)$	$O\left( n^4\right)$	|
Full Turner	$O\left( n^{|S|+3}\right)$	$O\left( n^5\right)$	|

### More realistic energy models

For the sake of simplicity, we illustrated in Sect. "Automatic derivation of dynamic programming equations in a base pair-based energy model" the generation of a dynamic programming algorithm within a fairly simple base-pair based energy model. However, the procedure can be adapted to capture more complex energy models found in the literature. This includes stacking base pairs models defined as:

$$\begin{aligned} E_{Stacking}(S) = \sum _{\begin{array}{c} \{(i,j),(i+1,j-1)\}\subset S \end{array}} \Delta G_{i,i+1,j-1,j} \end{aligned}$$

with $\Delta G_{i,i+1,j-1,j}$ the energy of base pair $(i+1,j-1)$ stacking onto (*i*, *j*), or even the nearest-neighbor free-energy model, also called Turner model.

In the Turner model, any pseudoknot-free structure *S* is decomposed into loops, each rooted at a base pair $(i,j)\in S\cup \{(-1,n+1)\}$, and delimited by a set of base pairs $L(i,j)=\{(i',j')\}\in S$ such that $[i',j']\subset [i,j]$ and $\not \exists (i'',j'' )\in S$ such that $[i',j']\subset [i'',j'' ]\subset [i,j]$. A loop *L*(*i*, *j*) is then assigned a free-energy contribution $\Delta G_{L(i,j)}$ that depends on the nucleotide content of base pairs, and unpaired regions between adjacent base pairs. The overall free energy of a structure in the Turner model is then defined as

$$\begin{aligned} E_{Turner}(S) = \sum _{(i,j)\in S\cup \{-1,n+1\}} \Delta G_{L(i,j)}. \end{aligned}$$

Rather than including independent values for all contents and size of loops, the Turner model usually uses affine linear models for multiloops ($|L(i,j)|\ge 2$), and interior loops ($|L(i,j)|=1$), the latter based on loop length and asymmetry.

Both of those models can be captured by a modified version of the dynamic programming algorithm presented in Sect. "Automatic derivation of dynamic programming equations in a base pair-based energy model". In the stacking model, it suffices to duplicate the cliques (resp. diagonal) matrices to keep track of (*i*, *j*) being directly enclosed ($\perp$) or not ($\not \perp$) within a base pair $(i+1,j-1)$. This results in a replacement $(C_\boxtimes ,C'_\boxtimes )$ with $(C_{\boxtimes ,\perp }, C'_{\boxtimes ,\perp },C_{\boxtimes ,\not \perp }, C'_{\boxtimes ,\not \perp })$ (resp. $(D_H,D'_H)$ into $(D_{H,\perp },D'_{H,\perp },D_{H,\not \perp },D'_{H,\not \perp })$), and the inclusion of suitable energy contributions for the $\perp$ cases, the only ones likely to form stacking pairs. The time complexity remains identical, up to a constant, to that of the BP energy model.

A consideration of the full Turner model is more involved, but can be achieved in $O(n^3)$ through an enumeration of all possible loops, as shown by Lyngsoe et al [39], by exploiting the linear interpolation of loops beyond a certain length threshold. Adapting the recurrence to consider all possible helix expansions of cliques and diagonals will result in a *O*(*n*) time overhead for all cliques and diagonals, leading to an increased time complexity in $O(|\gamma |\cdot n^{\max (w_{\boxtimes }+1,w_{\boxslash }+1,w'+1)})$, or equivalently $O(|\gamma |\cdot n^{tw+1})$. A summary of the complexity of filling the different kinds of DP table (transitional, clique and diagonal) depending on the choice of energy is given on Table 1. In any case, the space complexity is always $O(|\gamma |\cdot n^{tw})$, as stated below.

#### Lemma 3

The space complexity of the generated DP schedule is $O(|\gamma |\cdot n^{tw})$, regardless of the energy model.

#### Proof

The set of indices of a table is the intersection of the corresponding bag with its parent bag. Both bags have size at most $tw+1$, and they are distinct, so their intersection has size at most *tw*. Each index runs in the range [0, *n*], so the size of each table is at most $n^{tw}$. The number of tables is bounded by the number of bags in the tree decomposition of $\gamma$, which is itself in $O(|\gamma |)$. $\square$

### Partition functions and ensemble applications

For ensemble applications of our DP schemes, such as computing the partition function [40] and statistical sampling of the Boltzmann ensemble [41], it is imperative for the DP scheme above to be complete and unambiguous [42]. Fortunately, both properties are already guaranteed by our DP schemes. Indeed, intuitively: the completeness is ensured by the exhaustive investigation of all possible anchor positions, i.e. all possible partitions; the unambiguity is guaranteed by the invariant that assigning a position *x* to a given anchor (within a transitional or diagonal bag), leads *x* to be paired within the (half-)helix immediately to its right. Choosing different values for *x* thus induces different innermost/outermost base pairs for the associated helix, leading to disjoint sets of structures.

From these two properties, we conclude that the partition function for a fatgraph (or several, possibly recursively and/or within a ± realistic energy model) can be obtained through the simple change of algebra pioneered by McCaskill [40] in the pseudoknot-free case. Namely, replace the $(\min ,+,\Delta G)$ terms into $(\sum ,\times ,e^{\beta \Delta G})$, with $\beta =RT$ being the Boltzmann constant multiplied by some absolute temperature.

## Automated (re-)design of algorithms for specific pseudoknot classes

**Table 2:** Table listing pseudoknot classes, corresponding treewidth and resulting complexity of the folding algorithm

			Complexities	
Type	Fatgraph	Treewidth	Full Turner	All others
H-type	([)]	4	$O\left( n^{5}\right)$	$O\left( n^4\right)$(*)
Kissing hairpins	([)(])	4	$O\left( n^5\right)$	$O\left( n^4\right)$
“L” [12]	([{)]}	5	$O\left( n^{6}\right)$	$O\left( n^6\right)$
“M” [12]	([{)(]})	5	$O\left( n^{6}\right)$	$O\left( n^6\right)$
4-clique	([{<)]}>	5	$O\left( n^{6}\right)$	$O\left( n^6\right)$
5-clique	([{<A)]}>a	5	$O\left( n^6\right)$	$O\left( n^6\right)$
5-chain	({[)(][)}]	6	$O\left( n^{7}\right)$	$O\left( n^{7}\right)$

For H-type pseudoknots beneath the Turner model, marked as (*), an iterated computation over canonical tree decompositions is required to achieve the complexity (see Theorem 5). For the H-type and kissing hairpins cases, we are in the specific case where the most complex routine is the alignment of a “clique case” helix, which is done in $O(n^4)$ despite a treewidth of 4. These examples are detailed in the Appendix, Fig. 10. The DP equations for each of these examples have been automatically generated by a Python implementation of our pipeline, freely available at https://gitlab.inria.fr/bmarchan/auto-dp

Our pipeline for automated generation of DP folding equations given a fatgraph has been implemented using Python and Snakemake [43]. The implementation is freely available at: https://gitlab.inria.fr/bmarchan/auto-dp.

Since the algorithms in [12] have been described in terms of a finite number of fatgraphs (called irreducible shadows in the paper), one can directly apply our method to obtain an efficient algorithm that covers the same class as gfold, namely **1-structures** that are recursive expansions of the four fatgraphs of genus 1 corresponding to simple PK ’H’ ([)], kissing hairpin ’K’ ([)(]), three-knot ’L’ ({[)}] and ’M’ ([{)(]}) (here, represented in *dot-bracket notation*, i.e. corresponding opening and closing brackets correspond to arcs). The maximum complexity of $O(n^6)$ of the four fatgraphs (see Table 2) implies that the automatically derived algorithm covers the class of 1-structures in $O(n^6)$ time—the same complexity as hand-crafted gfold. Note that [12] used declarative methods in their algorithm design only to the point of generating grammar rules, which without further optimization yield $O(n^{18})$ (after applying algebraic dynamic programming; ADP [44]). In contrast, our method obtains the optimal complexity in fully automatic fashion. Beyond this re-design of gfold, remarkably our method is equally prepared to automatically design a DP algorithm with optimized efficiency for **2-structures**, which are based on all genus 2 fatgraphs. This is remarkable, since the implementation of a practical algorithm has been considered infeasible [12] due to the large number of genus 2 shadows (namely, there are 3472 shadows/fatgraphs), whose grammar rules would have to be optimized by hand. In contrast, due to full automation, our method directly handles even the large number of fatgraphs of genus 2 and yields an efficient, complexity optimized, DP scheme.

Recall that we cover all other pseudoknot classes that are recursive expansions of a finite number of fatgraphs (in the same way as we cover the design of prediction algorithms for 1- and 2-structures). In this way, among the previously existing DP algorithms, we cover the class of **Dirks &Pierce** (D &P) [11], simply by specifying the H-type as single input fatgraph. Consequently, we automatically re-design the D &P algorithm in the same complexity of $O(n^5)$. Even more interestingly, we can design algorithms covering specific (sets of) crossing configurations. This results in an infinite class of efficient algorithms that have not been designed before. Again the complexity of such algorithms is dominated by the most complex fatgraph; where results for interesting ones are given in Table 2. Most remarkably, we design an algorithm optimizing over recursive expansions of kissing hairpins in $O(n^4)$, whereas CCJ [13, 45], which was specifically designed to cover kissing hairpins, requires $O(n^5)$.

A special case, which further showcases the flexibility, is the extension of existing classes by specific crossing configurations. For example, extending D &P by kissing hairpin covers a much larger class while staying in the same complexity. Extending 1-structures by 5-chain yields a new algorithm with a complexity below of 2-structures (namely only $O(n^7)$ instead of $O(n^8)$ [12]). The complexity of 5-chain is remarkably low, when considering that previously described algorithms covering this configuration take $O(n^8)$ (e.g. gfold’s generalization to 2-structures and a hypothetical blow-up of the Rivas and Eddy algorithm [10] to 6-dimensional instead of 4-dimensional DP matrix elements—both of which have never been implemented).

## Conclusions and discussion

In this work, we provided an algorithm that takes a family of fatgraphs, i.e. pseudoknotted structures, and returns DP equations that efficiently predict arc annotations minimizing the free energy. The DP equations are automatically generated based on an expansion of the fatgraph, designed to capture helices of arbitrary length. The DP tables in the equations use a number of indices smaller than or equal to the treewidth of the minimal expansion. This very general framework recovers the complexity of prior, hand-crafted algorithms, and lays the foundation for a purely declarative approach to RNA folding with pseudoknots.

In addition to the extensions described in Sect. "Extensions", this work suggests perspectives that will be explored in future work. Indeed, the choice of an optimal decomposition/DP scheme for the input fatgraph can be seen as the automated design of an optimal table strategy in the context of algebraic dynamic programming [44, 46, 47]. This would enable extensions to multiple context free grammars or tree grammars when describing the problem in the ADP framework.

Our automated design of pseudoknot folding algorithms could naturally be extended to RNA–RNA interactions, since the joint conformation of two interacting RNA sequences can be seen as a pseudoknot when concatenating the two structures [48]. More ambitiously, categories of pseudoknots inducing an infinite family of fatgraphs, e.g. as covered by the seminal Rivas & Eddy algorithm [10], could be captured by allowing the introduction of recursive gapped structures in prescribed parts of the fatgraph. This could be addressed by adding cliques to the minimal completion graph which would ensure the availability of the relevant anchors in some bags of the tree decomposition, allowing to score such non-contiguous, recursive substructures.

Another avenue for future research includes a proof of optimality, in term of polynomial complexity, for the produced DP algorithms. Of course, it would be far too ambitious (and erroneous) to expect our DP schemes to be optimal within general computational models. However, it may be possible to prove optimality within a formally-defined subset of DP schemes, e.g. by contradiction since the existence of a better algorithm would imply the existence of a tree decomposition having smaller width. More precisely, given a fatgraph $\gamma$, one could imagine that a DP scheme (with DP tables indexed by *anchor variables* as is typically the case) capable of exploring all recursive expansions of $\gamma$ would in particular induce a *decomposition* of the minimal representative expansion of $\gamma$, from the *parsing* of this structure by the DP grammar. If this decomposition can be reinterpreted as a tree decomposition, then the treewidth of the minimal expansion would become a lower bound on the number of indices to use in such a DP scheme.

## Data Availability

A prototype implementation of our algorithm is available at https://gitlab.inria.fr/bmarchan/auto-dp

## Appendix

See Figs. 10, 11
